# Genetic Analysis of the Salmonella FliE Protein That Forms the Base of the Flagellar Axial Structure

**DOI:** 10.1128/mBio.02392-21

**Published:** 2021-09-28

**Authors:** Jordan J. Hendriksen, Hee Jung Lee, Alexander J. Bradshaw, Keiichi Namba, Fabienne F. V. Chevance, Tohru Minamino, Kelly T. Hughes

**Affiliations:** a School of Biological Sciences, University of Utah, Salt Lake City, Utah, USA; b Graduate School of Frontiers Bioscience, Osaka University, Suita, Osaka, Japan; c RIKEN Center for Biosystems Dynamics Research and SPring-8 Center, Suita, Osaka, Japan; d JEOL YOKOGUSHI Research Alliance Laboratories, Osaka University, Suita, Osaka, Japan; The Ohio State University

**Keywords:** flagellum, FliE, genetics, *Salmonella*, structure, type 3 secretion

## Abstract

The FliE component of the bacterial flagellum is the first protein secreted through the flagellar type III secretion system (fT3SS) that is capable of self-assembly into the growing bacterial organelle. The FliE protein plays dual roles in the assembly of the Salmonella flagellum as the final component of the flagellar type III secretion system (fT3SS) and as an adaptor protein that anchors the rod (drive shaft) of the flagellar motor to the membrane-imbedded MS-ring structure. This work has identified the interactions between FliE and other proteins at the inner membrane base of the flagellar machine. The *fliE* sequence coding for the 104-amino-acid protein was subject to saturating mutagenesis. Single-amino-acid substitutions were generated in *fliE*, resulting in motility phenotypes. From these mutants, intergenic suppressor mutations were generated, isolated, and characterized. Single-amino-acid mutations defective in FliE function were localized to the N- and C-terminal helices of the protein. Motile suppressors of amino acid mutations in *fliE* were found in rod protein genes *flgB* and *flgC*, the MS ring gene, *fliF*, and one of the core T3SS genes, *fliR*. These results support the hypothesis that FliE acts as a linker protein consisting of an N-terminal α-helix that is involved in the interaction with the MS ring with a rotational symmetry and a C-terminal coiled coil that interacts with FliF, FliR, FlgB, and FlgC, and these interactions open the exit gate of the protein export channel of the fT3SS.

## INTRODUCTION

Salmonella enterica is estimated to cause illness in over 1 million people annually in the United States. Inside a host, Salmonella uses an organelle known as the flagellum for both motility (swimming) and pathogenesis (infection). The majority of the flagellar organelle is extracellular, and a type III secretion system (T3SS) secretes structural subunits from the cytoplasm to be assembled into the growing structure (1, 2). Salmonella enterica possesses two other T3SSs that are evolutionarily related to the flagellar T3SS (fT3SS) (3). The other T3SSs are involved in the secretion of virulence-associated effector proteins into host cells to facilitate Salmonella pathogenesis. These effector proteins are secreted through hypodermic needle-like structures called injectisomes (4).

**FIG 1:**
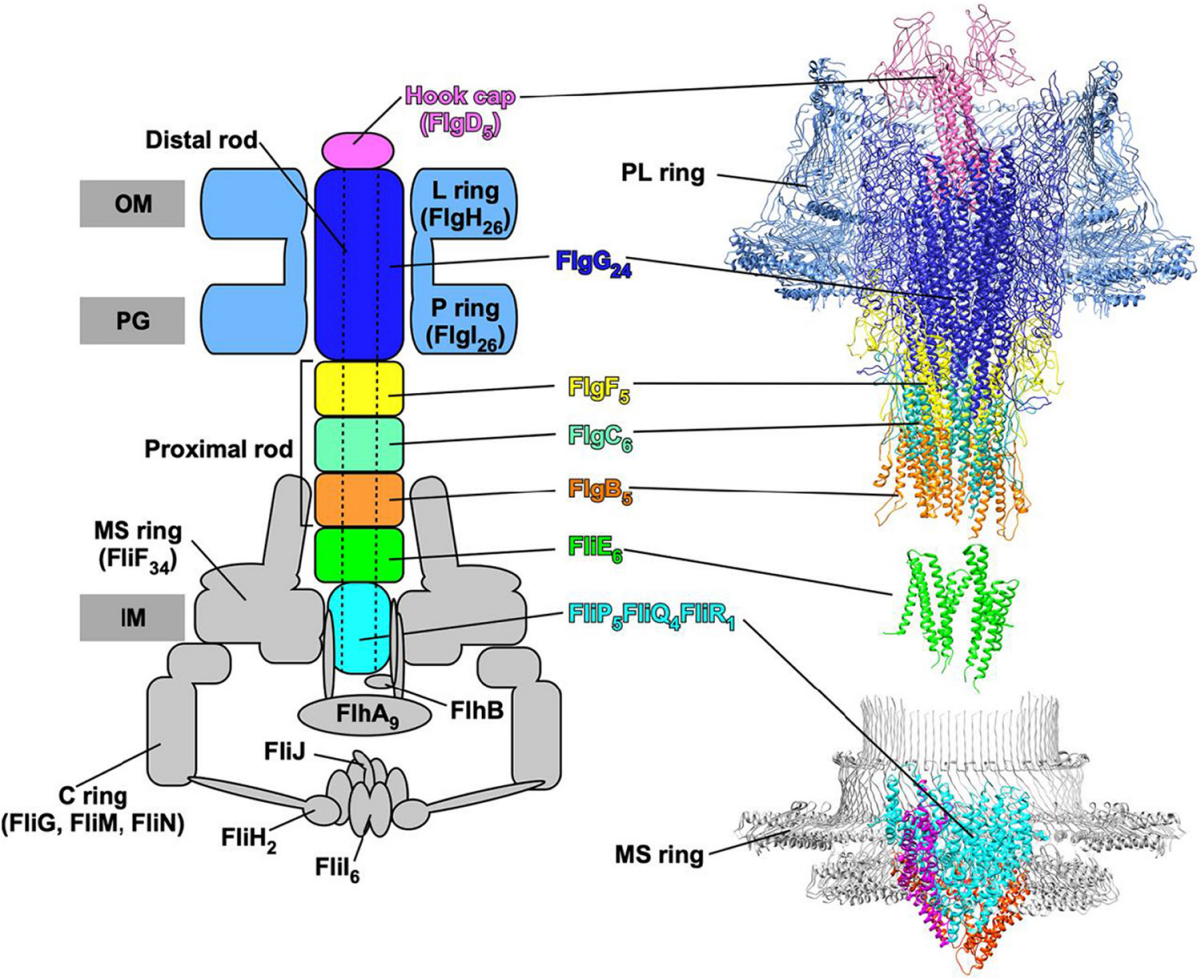
CryoEM structure of the Salmonella basal body refined in C1 map (PDB entry 7NVG) (left) and its schematic diagram (right). The basal body is composed of the MS-ring (FliF), C-ring (FliG, FliM, and FliN), P-ring (FlgI), L-ring (FlgH), and rod (FliE, FlgB, FlgC, FlgE, and FlgG). The cryoEM structure for the basal structure, including the MS-ring, rod, and PL-ring, has recently been solved (18, 19), as has the FliP_5_FliQ_4_FliR core secretion complex (13). The flagellar type III secretion system (fT3SS) is located at the flagellar base and transports flagellar building blocks such as rod proteins from the cytoplasm to the distal end of the growing flagellar structure. The fT3SS is composed of a transmembrane export gate complex made of FlhA, FlhB, FliP, FliQ, and FliR and a cytoplasmic ATPase ring complex consisting of FliH, FliI, and FliJ. The export gate complex is located inside the MS ring. FliP, FliQ, and FliR form a protein export channel complex with a stoichiometry of 5 FliP, 4 FliQ, and 1 FliR, with a helical symmetry similar to that of the rod. FliE binds to FliP and FliR to open the exit gate of the polypeptide channel. FlhB associates with the FliP_5_FliQ_4_FliR_1_ complex and coordinates gate opening for substrate entry into the protein export channel. FlhA assembles into a homononamer and acts as an export engine. IM, cytoplasmic membrane; PG, peptidoglycan layer; OM, outer membrane.

The bacterial flagellum is a complex structure that utilizes more than 30 gene products for its construction (5). The genes that encode Salmonella flagellar proteins are named based on the chromosomal locations where they are located: the *flg*, *flh*, *fli*, and *flj* regions (6). The flagellum is generally divided into three main structural components: the basal body, the hook, and the filament. The basal body serves as the ion-powered rotary motor that anchors the structure within the cell membrane(s) and cell wall (Fig. 1) (7).

Flagellum assembly begins at the cytoplasmic membrane with the formation of the MS-ring that is composed of ∼34 subunits of a single protein, FliF (8, 9). Beneath the MS-ring is a cytoplasmic C-ring structure that acts as the rotor of the flagellar motor. Within the MS-ring, a transmembrane export gate complex of the fT3SS forms, which is composed of FlhA, FlhB, FliP, FliQ, and FliR (10), and for which structural components have been solved (11–13). The rod structure acts as a drive shaft that extends from the MS-ring fT3SS base and transverses the periplasmic space through the cell wall to the outer membrane. A bushing, known as the PL-ring, assembles around the tip of the rod and forms a pore in the outer membrane. An ∼55-nm hook extends from the cell surface to which the long external filament grows to ∼10 μm. The hook acts as a universal joint between the rigid rod and filament, allowing rotation of the flagellum extending from either cell pole to propel the bacterium.

The MS-ring is planar in the inner membrane, yet the rod extends from an axial structure akin to a spiral staircase from the fT3SS base within the MS-ring. The connection of these geometrically different structures at the base is accomplished with the assembly of the FliE protein. The FliE protein has dual roles in the flagellar assembly process: (i) as a structural component that anchors the axial rod-hook-filament to the inner membrane-embedded MS-ring-fT3SS (14) and (ii) as the final component of the fT3S system (10, 15). However, unlike FliF, FlhAB, and FliPQR, FliE does not have a homolog in the injectisome-associated T3S systems based on amino acid identity. However, the location of SctI in the injectisome structure suggests that FliE is a functional homolog of SctI (16). The fact that the flagellum rotates at speeds of 300 to 2,000 revolutions per second while the injectisome is a static hypodermic needle-like structure may be the reason FliE and SctI lack homology. FliE is also of interest for structural reasons in that it is does not share homologous sequences with the axial proteins, which share homologous sequences with each other (14). FliE is 104 amino acids in length. Recent work suggests it has a functional domain within the first 18 amino acids followed by a spacer region of ≥15 amino acids and at least one other functional domain after amino acid 33. This conclusion is based on the isolation of motile revertants of a nonfunctional *fliE* mutant deleted for amino acids 18 to 31 (17). Motile revertants of this deletion mutant resulted from tandem duplications of *fliE* sequences flanking the deleted sequences that restored the length of the protein to near that of wild-type FliE. Recently, the Salmonella basal body structure has been solved by cryoelectron microscopy (cryoEM) image analysis (Fig. 1) (18, 19). Six copies of FliE assemble into the most proximal part of the rod in the MS-ring. FliE consists of three α-helices, α1, α2, and α3. The α1 helix binds to the inner wall of the MS-ring. The α2 and α3 helices form domain D0 in a way similar to other rod proteins. The D0 domain of FliE interacts with FliP, FliR, FlgB, and FlgC in the basal body.

In the present study, we sought to elucidate the role of FliE as the anchor for the axial rod-hook-filament to the inner membrane-embedded fT3SS components. We were also able to take advantage of recent published work on the atomic structures of the flagellar basal body and T3S complex in order to characterize the interactions between FliE, the MS-ring (FliF), and components of the core T3S complex. To this end, we divided *fliE* into segments for targeted mutagenesis that were screened separately for single-amino-acid substitutions defective in flagellar secretion and assembly. Missense alleles in *fliE* were then used to isolate second-site suppressor alleles in *flgB*, *flgC*, *fliF*, and *fliR*, which restored motility as a means to identify components of the secretion apparatus that interacted with FliE. We also isolated suppressors of strains deleted for sequences in regions including amino acids 15 to 28, 18 to 31, and 21 to 39 that resulted from tandem duplications of *fliE* sequences. The results of this study support the model that FliE has N- and C-terminal functional domains: missense alleles were only obtained in the first 20 and last 30 amino acids of FliE. Extragenic motile suppressors of *fliE* missense alleles suggest that intermolecular packing interactions of domain D0 of FliE with the FlgB and FlgC proximal rod proteins stabilize the open conformation of a protein export channel to form a continuous path for one-dimensional diffusion of flagellar axial proteins into the distal end of the growing structure.

## RESULTS

### Isolation of *fliE* mutant alleles.

We sought to isolate *fliE* mutants resulting from single-amino-acid substitutions that produced a strong motility defect, which would facilitate the isolation of second-site suppressor mutations. To date, only one such substitution in FliE, V99G, has been reported and was used to isolate extragenic suppressors in the *flgB* gene (20). This provided evidence for an interaction between FliE and FlgB and that FlgB was the first axial rod protein assembled into the flagellar basal body. PCR-directed mutagenesis of the entire *fliE* coding sequence followed by DNA sequence analysis on nonmotile isolates allowed us to obtain seven mutant alleles of *fliE* that were not the result of nonsense or frameshift mutations. These included single-amino-acid substitutions, L38P, L61P, V99A, and S101P, and three *fliE* alleles with multiple-amino-acid substitutions D74G S79P, Q13R H27R F59S, and G65D M82T M84T. We did not obtain the V99G substitution described earlier, indicating that the mutagenesis of *fliE* was not saturated. The D74G and S79P substitutions were separated by site-directed mutagenesis (see Materials and Methods). The D74G substitution alone exhibited the same motility as the wild type (*fliE*^+^) (see Fig. S1A in the supplemental material) while the S79P substitution exhibited the same motility defect as the double mutant at 37°C, but at 30°C the D74G S79P double substitution mutant was significantly less motile than the S79P single substitution (Fig. S1B). Site-directed mutagenesis was also employed to construct single-amino-acid substitutions in *fliE*: G65D, M82T, and M84T. The individual substitutions exhibited wild-type motility (Fig. S2).

10.1128/mBio.02392-21.1 FIG S1 (A) Motility assay of the *fliE* single (D74G or S79P) and double (D74G S79P) substitution alleles, the individual *fliF* N209D suppressor allele of the *fliE* D74G S79P double mutant allele compared to LT2 wild-type (*fliE*^+^) and a *fliE* deletion mutant (Δ*fliE*) at 37°C. (B) The *fliE* D74G S79P double substitution and the and the *fliE* S79P single substitution mutants exhibited some and significant motility, respectively, compared to wild-type motility at 30°C. The D74G single substitution mutant had wild-type motility at 30°C (not shown). Download FIG S1, DOCX file, 0.2 MB. Copyright © 2021 Hendriksen et al. 2021 Hendriksen et al. https://creativecommons.org/licenses/by/4.0/ This content is distributed under the terms of the Creative Commons Attribution 4.0 International license.

10.1128/mBio.02392-21.2 FIG S2 (A) Motility assay of the *fliE* G65D M82T M84T triple substitution mutant without and with (Mot^+^) the *fliR* A83V suppressor allele compared to LT2 wild type (*fliE*^+^) and an *fliE* deletion mutant (Δ*fliE*) at 37°C. (B) Motility assay of individual *fliE* G65D, *fliE* M82T *fliE* M84T substitutions, and the *fliE* G65D M82 double substitution compared to the *fliE* G65D M82T M84T triple substitution mutant, the LT2 wild-type (*fliE*^+^) and an *fliE* deletion mutant (Δ*fliE*) at 37°C. (C) Motility assay of the individual *fliR* A83V allele compared to LT2 wild-type (*fliE*^+^) and a *fliE* deletion mutant (Δ*fliE*) at 37°C. Download FIG S2, DOCX file, 0.2 MB. Copyright © 2021 Hendriksen et al. 2021 Hendriksen et al. https://creativecommons.org/licenses/by/4.0/ This content is distributed under the terms of the Creative Commons Attribution 4.0 International license.

The isolation of *fliE* mutants described above was labor-intensive; following mutagenesis, colonies were screened individually for loss of motility. To facilitate the isolation of *fliE* mutants, we took advantage of aspects of flagellar gene regulation where expression of the flagellar filament genes is dependent on a functional FliE protein. The flagellar regulon is organized into a transcriptional hierarchy of three promoter classes, allowing flagellar gene expression to be coupled to flagellum assembly (21). At the top of this hierarchy is the class 1 *flhDC* operon. Transcription of *flhDC* depends on a variety of environmental signals. The FlhD and FlhC proteins form the multimeric FlhD_4_C_2_ transcriptional activator complex that binds flagellar class 2 promoter sequences to direct σ^70^-RNA polymerase-dependent transcription. The flagellar class 2 gene products include all the proteins necessary for the structure and assembly of the hook-basal-body structure. Also expressed from class 2 promoters are two key regulatory genes, *flgM* and *fliA*. The *fliA* gene encodes a flagellum-specific transcription factor, σ^28^, that directs RNA polymerase to transcribe from class 3 promoters. Class 3 genes encode the flagellar filament proteins FliC and FljB and genes of the chemosensory system. The FlgM protein is an anti-σ^28^ factor that prevents σ^28^ from interacting with RNA polymerase and will actively dissociate σ^28^ from RNA polymerase. Upon HBB completion, there is a secretion specificity switch in the fT3SS from early, rod-hook protein specificity to late substrate specificity. FlgM is a late secretion substrate that is secreted upon HBB completion. With FlgM removed from the cytoplasm, σ^28^ is free to direct RNA polymerase-dependent transcription at class 3 promoters. If any HBB substrate gene, including *fliE*, is defective, FlgM is not secreted and class 3 genes are not expressed.

Using a transcriptional fusion of the *lac* operon to the class 3 *fljB* gene, *fljB*::Mu*d*J, we could readily screen for mutants in any gene required for HBB assembly, including *fliE*. The *fljB*::Mu*d*J fusion confers a Lac^+^ phenotype on strains with a functional *fliE* gene and a Lac^−^ phenotype on strains defective in *fliE*. On tetrazolium-lactose (Tz-Lac) indicator medium, *fliE*^+^ *fljB*::Mu*d*J colonies are white and *fliE* mutant *fljB*::Mu*d*J colonies are dark red. Cells require <30 Miller units of β-galactosidase (β-Gal) to show a strong Lac^−^ phenotype on Tz-Lac (dark red) and >150 Miller units to show a strong Tz-Lac white (Lac^+^) phenotype (22). Furthermore, the use of lactose indicator plates allows for the screening of *fliE* alleles with partial activities showing intermediate color phenotypes: light pink to pink to light red to red on Tz-Lac plates.

The N (amino acid codons 2 to 19)- and C (amino acid codons 87 to 104)-terminal regions of *fliE* were initially targeted for “doped” oligonucleotide mutagenesis. The doped *fliE* oligonucleotide was synthesized where the chemical mixture for each wild-type base contained a small amount of the three other bases such that, on average, each *fliE* oligonucleotide contained a single random base substitution mutation throughout the coding sequence being targeted for mutagenesis. Twenty-five mutants with a strong *fliE* mutant (dark red colonies on Tz-Lac) nonmotile phenotype were analyzed by DNA sequencing, and all were due to either nonsense or frameshift mutations. We presumed that the frameshift mutations would have arisen by errors in oligonucleotide synthesis, suggesting that *fliE* null alleles resulting from single-amino-acid substitutions are rare. For this reason, in subsequent experiments, mutants with some apparent FliE function based on Tz-Lac indicator phenotypes were chosen for DNA sequencing.

In an attempt to saturate the mutagenesis of the entire *fliE* gene, six coding segments of *fliE* were independently targeted for mutagenesis. The following *fliE* coding regions were separately deleted and replaced with tetracycline (Tc) resistance, *tetRA*, cassettes from transposon Tn*10*: amino acids 2 to 19, 20 to 37, 38 to 54, 55 to 73, 74 to 86, and 87 to 104. Resistance to tetracycline (Tc^r^) provides a positive selection for integration of the *tetRA* cassette into a recipient chromosome. An advantage of using *tetRA* cassettes for targeted mutagenesis is that removal of the *tetRA* cassette can be positively selected for because strains carrying a *tetRA* cassette are sensitive to the lipophilic agent fusaric acid and do not grow on Tc-sensitive (Tc^s^) selection plates, which contain fusaric acid (23, 24). Each *tetRA* cassette was replaced by λ-Red recombination with doped oligonucleotide sequences. The targeted strains also carried a *lac* operon fusion to the σ^28^-dependent *fljB* promoter (*fljB*::Mu*d*J).

**FIG 2:**
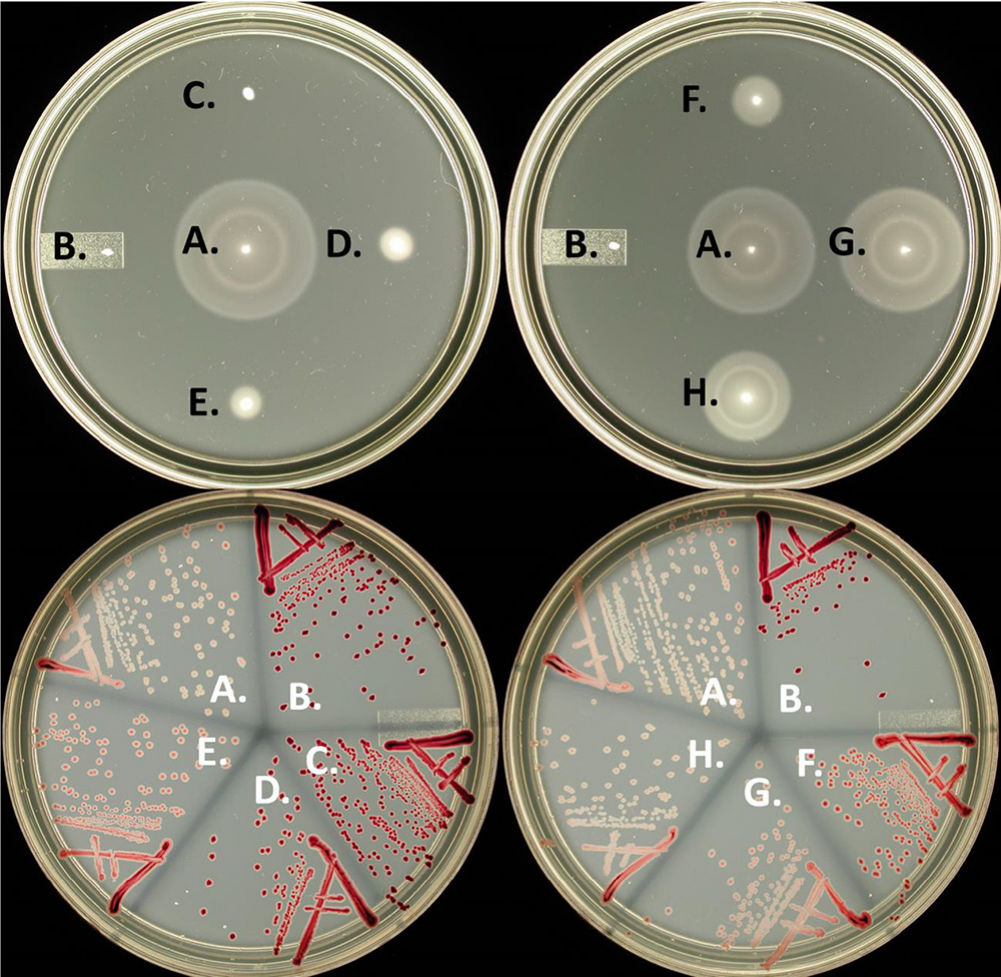
Effect of *fliE* mutations on motility and σ^28^-dependent class 3 transcription. Motility (top) and expression of a *fljB-lac* operon reporter construct using Tz-lactose indicator plate (Tz-Lac) (bottom) assays of *fliE* mutations with (left) and without (right) addition of one of their respective suppressor mutations. (A) Wild-type *fliE^+^*. (B) Δ*fliE* (gene deletion). (C) *fliE*(ΔR53-A56). (D) *fliE*(G85R). (E) *fliE*(Q103K). (F) *fliE*(ΔR53-A56) *flgC*(T105M). (G) *fliE*(G85R) *flgB*(G129D). (H) *fliE*(Q103K) *flK*(Q208^UAG^).

**FIG 3:**
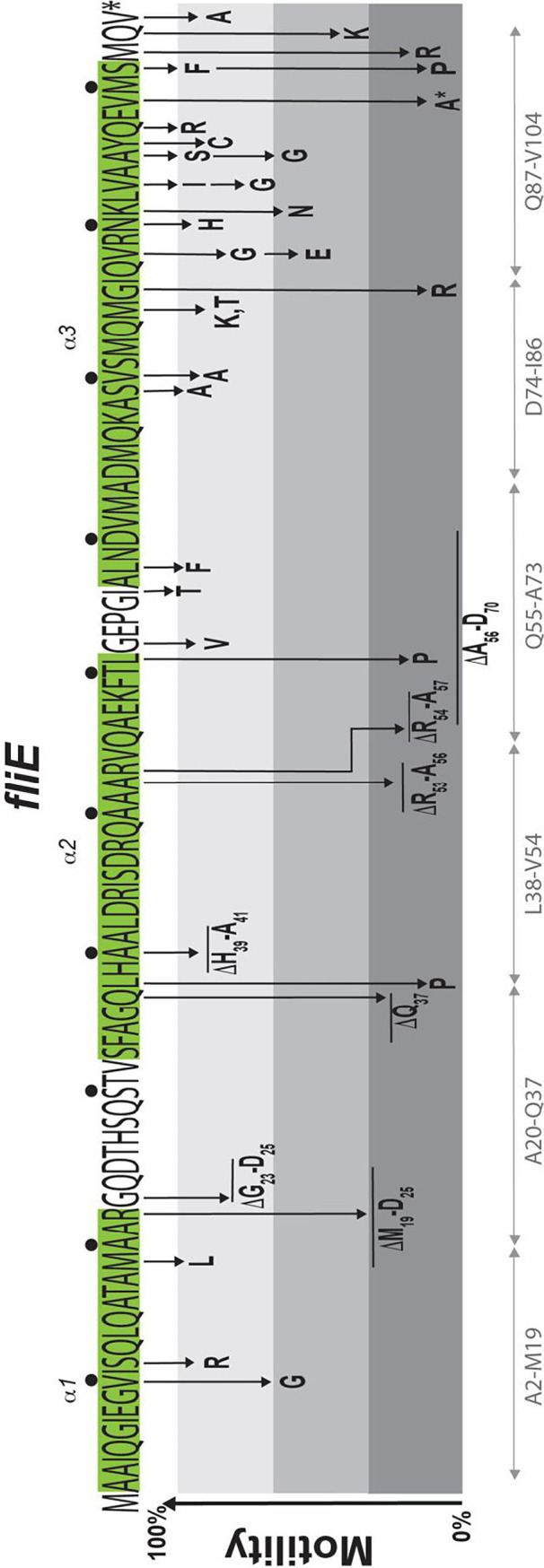
FliE sections and a subset of mutations showing location and motility phenotype. Sections mutagenized by doped oligonucleotide mutagenesis are shown across the *x* axis. The *y* axis is the phenotype as the approximate percentage of the wild type. The V99A (*) substitution was obtained by PCR mutagenesis of the *fliE* coding sequence only.

Using doped oligonucleotide mutagenesis, mutants in *fliE* were isolated that exhibited a range of FliE activities, as indicated on Tz-Lac plates, from dark red (slightly less dark red than the Δ*fliE* control strain) to light pink. The *fliE* mutants were also screened for behavior on motility plates. Colony color phenotypes on Tz-Lac plates correlated with motility on soft-agar swim plates, as shown in Fig. 2. Table 1 summarizes the results of the 30 single-amino-acid substitutions and two in-frame deletions (ΔQ37 and ΔR53-A56) in *fliE* compared to *fliE*^+^ with respect to motility at 30°C and their effect on *fljB-lac* expression on Tz-Lac indicator plates. Motility at 37°C was similar for all alleles except L61P and G85R, which exhibited 38% and 13% motility compared to the wild type at 37°C. With some exceptions, a reduction in motility correlated with a darker color phenotype on Tz-Lac plates. A total of 98 *fliE* mutants have been isolated and sequenced in the doped oligonucleotide mutagenesis study; many were isolated multiple times. The locations of the different substitutions and deletions within the *fliE* gene and the degrees of their effect on motility in soft-agar tryptone plates compared to the *fliE*^+^ parent strain are shown in Fig. 3. The 30 single-amino-acid substitutions were all found within the first 20 or last 45 amino acids of the protein (Table 1 and Fig. 3). All mutations isolated within the middle 40 amino acids contained at least one amino acid deletion. Significantly, no single-amino-acid substitutions between codons M19 and F59 were isolated by doped oligonucleotide mutagenesis, and none created substantial defects in function until codon G85. Substitutions L38P and L61P were isolated following PCR mutagenesis and screening for a strong nonmotile phenotype (0% of wild-type motility). Our working model, based on the mutagenesis results, is that the region between M19 and G85 functions primarily as a spacer region, which can tolerate any single-amino-acid substitution, that connects the N- and C-terminal regions, which contain critical residues for FliE function.

**TABLE 1:** Motility and flagellar class 3 expression phenotypes of *fliE* alleles

*fliE* mutation	Tz-Lac phenotype	Wild-type motility^*a*^ (%)
FliE^+^ control	White	100
ΔFliE (null control)	Dark red	0
V10G	Red	43
S12R	Red	67
M19L	Light pink	91
ΔQ37	Dark red	20
L38P	Dark red	0
ΔR53-A56	Dark red	27
A56A; A67P	Pink	93
F59S	Red	83
L61P	Dark red	38
G62V	Dark pink-light red	77
I66T	Dark pink	87
L68F	Pink	87
S79A	Dark pink	100
V80A	Dark pink	77
M84T	Dark pink	86
M84K	Dark pink-light red	87
G85R	Dark pink-light red	54
V88E	Red	81
V88G	Dark pink	87
N90H	Pink	89
K91N	Pink	86
V93I	Pink	94
V93G	Dark pink	82
A95S	Pink	79
A95G	Pink	73
Y96C	Red	75
Q97R	Pink	80
S101F	Light pink	86
S101P	Dark red	0
M102R	Dark red	23
Q103K	Red	31
V104A	Dark pink	77

a Motility at 30°C relative to the wild type is presented.

### Suppression of *fliE* in-frame deletions by adjacent sequence duplication.

**FIG 4:**
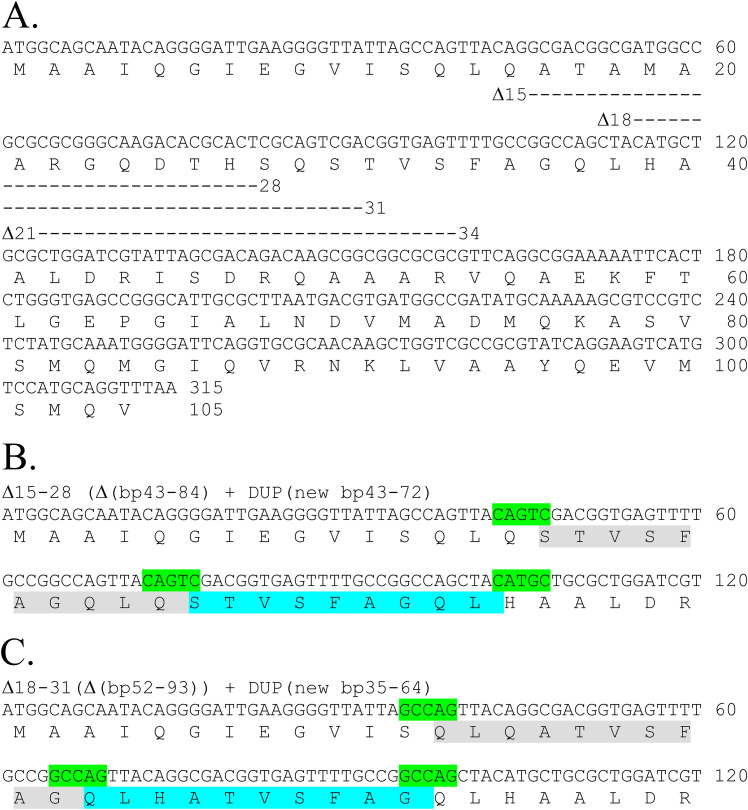
Motile suppressors of internal *fliE* deletion mutants. (A) The DNA and amino acid sequence of the *fliE* gene and translated protein indicating the positions of internal deletions used in this study. (B) A motile suppressor of the *fliE* deletion lacking amino acids 15 through 28 resulted from a duplication of 30 bp flanking 3′ to the deletion endpoint. (C) A motile suppressor of the *fliE* deletion lacking amino acids 18 through 31 resulted from a duplication of 30 bp flanking 5′ to the deletion endpoint. Both duplication suppressor alleles result in a predicted 100-amino-acid FliE protein.

Recently, Sasias et al. (17) reported that 4 out of 10 human clinical isolates of Salmonella serovar Dublin they characterized were nonmotile due to an in-frame deletion of codons 18 through 31 in *fliE*. Motile revertants of the in-frame *fliE* deletion were obtained and resulted from internal duplications of DNA bases in *fliE* that somewhat restored the spacing between N- and C-terminal FliE regions. The duplications resulted in functional FliE proteins of 100 and 107 amino acids in length, whereas the wild-type FliE protein is 104 amino acids in length. We repeated their experiment in Salmonella Typhimurium by constructing in-frame deletions in *fliE* lacking 14 codons for amino acids 15 to 28, 18 to 31, or 21 to 34 and selecting for motile revertants of these *fliE* deletion mutants. For 10 independent plating experiments, the *fliE* mutant deleted for codons 21 to 34 [Δ(21-34)] failed to yield motile revertants after 5 days of incubation at 37°C, while the Δ(15-28) and Δ(18-31) mutants were able to mutate to Mot^+^. The Mot^+^ revertant from the Δ(15-28) (deletion of base pairs 43 to 84) allele appears to result from a recombination event between 5 bases, giving rise to a 30-base duplication (Fig. 4). The Mot^+^ revertant from the Δ(18-31) allele also appears to result from a recombination event between 5 bases giving rise to a 30-base duplication. Both revertant duplications resulted in a 100-amino-acid FliE protein. These results support a model where N- and C-terminal regions of FliE are critical for function provided they are properly spaced from each other.

### Mutations in *flgB*, *flgC*, *fliF*, *fliR*, and *flk* suppress motility defects of *fliE* mutant alleles.

A previous study reported that the motility defect of an *fliE* V99G missense mutant was suppressed by single-amino-acid substitutions, either G119E or G12D in FlgB. This led to a model of rod assembly where FliE is incorporated first, followed by FlgB (20). This result agrees with later work on Borrelia burgdorferi flagellum assembly using cryoelectron tomography in support of a proximal rod subunit order of assembly FliE-FlgB-FlgC-FlgF (25). In this study, an *fliE* mutant showed no rod, whereas mutants in *flgB*, *flgC*, and *flgF* showed increased rod lengths, which is consistent with the assembly of FliE followed by FlgB, FlgC, and then FlgF. Since we have obtained a plethora of new mutants in *fliE*, including single-amino-acid substitutions and small deletions, we sought unlinked motile suppressors in order to identify other subunits of the basal body that might contact FliE. Mutants with a substantial motility defect were selected to generate suppressor alleles, including ΔR53-A56, (D74G, S79P), (G65D, M82T, M84T), G85R, and Q103K. Motile revertants of these alleles were isolated and purified from motile flairs that arose on motility plates after prolonged incubation of the motility-defective alleles. Phage P22-mediated transduction using markers linked to the *flg*, *flh*, *fli*, and *flj* flagellar gene regions was used to determine the location of the suppressor mutations. Suppressor alleles generated by ΔR53-A56 and G85R mapped to the *flg* region, and suppressors of the (D74G, S79P) double mutant and (G65D, M82T, M84T) triple mutant mapped to the *fli* region of the chromosome. DNA sequence analysis revealed three suppressors within the *flg* locus. A motile suppressor of the *fliE* ΔR53-A56 allele resulted from a T105M substitution in *flgC*. Two independent substitutions in *flgB* (G119E and G129D) were found to suppress the motility defect of the *fliE* G85R mutant. When moved into an *fliE*^+^ background, the T105M substitution in FlgC had no motility defect. In contrast, both *flgB* suppressor alleles of the FliE G85D mutant, FlgB G119E and FlgB G129D, have a small but measurable impact on motility, exhibiting decreased swarm sizes on motility plates to 89% and 65%, respectively, of the wild type (Table 2). The motile suppressor of the triple substitution mutant in *fliE* (D74G, M82T, M84T) resulted from an A83V substitution mutation in *fliR*. The FliR protein is a component of the core secretion pore complex (13, 26).

**TABLE 2:** Intergenic motile suppressors of specific *fliE* mutants

Strain no.	Intergenic suppressor mutations		
	FliE substitution(s)	Suppressor mutation	Motility of isolated suppressor alleles (% of WT)
TH11373	D74G S79P	*fliF7059*(N209D)	See Fig. S1
TH25994	Q103K	*flk-8905*(W54:UAG)	100
TH25995	Q103K	*flk-8906*(Q208:UAG)	100
TH25996	Q103K	Δ(*pdxB*-*flk*)*-8906*	100
TH25992	G85R	*flgB8903*(G119E)	89
TH25993	G85R	*flgB8904*(G129D)	65
TH24121	ΔR53-A56	*flgC8647*(T105M)	100
TH11374	G65D, M82T, M84T	*fliR7060*(A83V)	100

Motile revertants arising from *fliE* Q103K were unlinked to the *fli*, *flg*, *flh*, or *flj* chromosomal flagellar gene regions. However, while checking linkage, it was noticed that one motile revertant was unable to grow on minimal medium. Screening this mutant for growth on various auxotrophic supplemental pools (27), it was determined that reversion to motility revertant had resulted in pyridoxine auxotrophy. A flagellar gene outside the common loci, *flk*, had been shown to share an overlapping divergent promoter with a gene required for pyridoxine synthesis, *pdxB* (28). Analysis of the *flk*-*pdxB* region by PCR determined that the *fliE* Q103K suppressor mutation resulted from deletion of both *flk* and *pdxB.* Subsequent sequencing of additional Q103K motile revertants determined them to be null alleles in *flk*, including stop codons at positions W54 and Q208.

**FIG 5:**
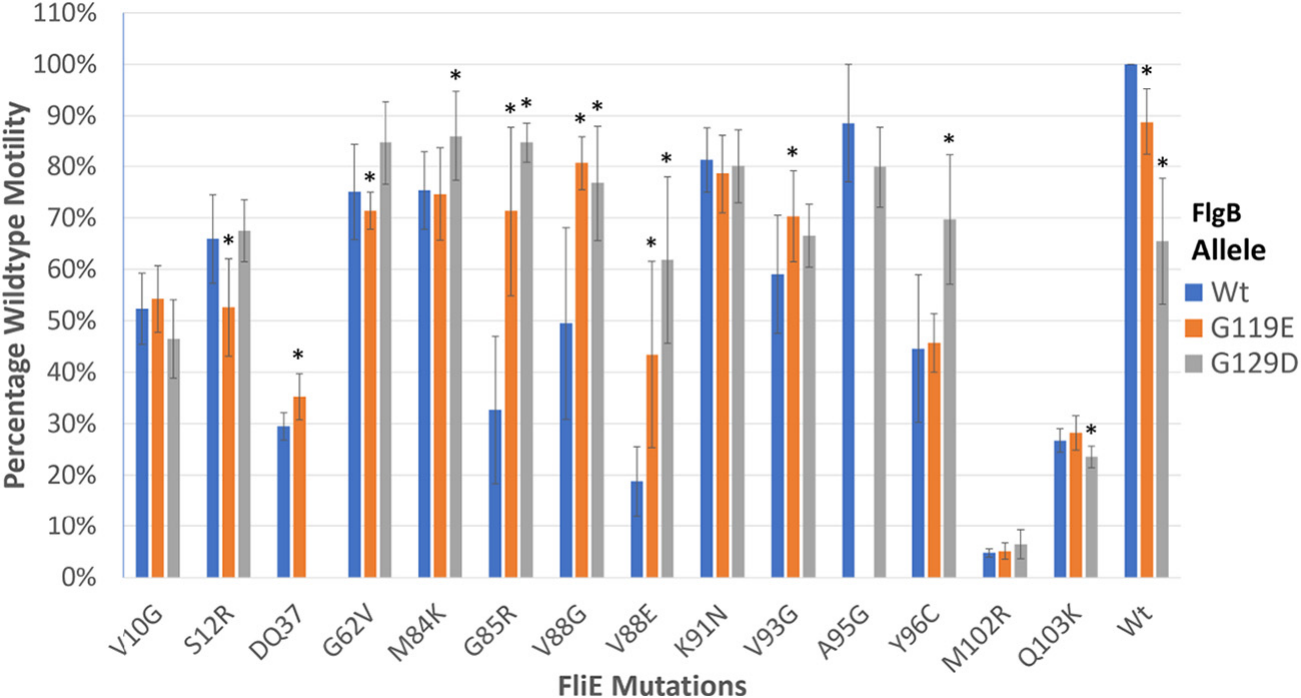
Column graph of the relative motility of the different combinations of FliE and FlgB alleles. The *y* axis is the percentage of wild-type motility, and the *x* axis is the *fliE* substitution allele found within that set of combinations. Colors denote the *flgB* allele found within the strains: blue is a wild-type allele, orange is *flgB*(G119E), and gray is *flgB*(G129D). Each column has error bars representing the standard deviation of that combination’s motility. An asterisk identifies the combinations that have a statistically significant change in motility (*P* < 0.05) (*n* = 8) compared to their *fliE* allele combined with a wild-type *flgB* allele (blue columns).

The *flgB* suppressor alleles, G119E and G129D, previously have been described to suppress the motility defect caused by *fliE* V99G (20). Given that these same suppressor mutations suppress the motility defect of *fliE* G85R, it was suspected that the region of FliE including codons G85-V99 interact with the C-terminal portion of FlgB between codons G119 and G129. To define this region of interaction, strains were constructed combining each *flgB* allele with one of 14 mutant *fliE* alleles. The motility of each of these combinations was characterized with 8 replicates. Two-tailed Student’s *t* tests, with α of 0.05, comparing the *fliE* mutants with a wild-type *flgB* allele to that same mutant with a suppressor *flgB* allele are summarized in Fig. 5 and Table S3. Motility defects caused by ΔQ37, G85R, V88G, V88E, and V93G were significantly suppressed by *flgB* G119E. Defects caused by M84K, G85R, V88G, V88E, and Y96C were significantly suppressed by *flgB* G129D and to a greater extent than the suppression resulting from *flgB* G119E. Both *flgB* alleles caused a significant motility defect when paired with a wild-type *fliE* allele. In addition, the FlgB G119E substitution also caused a defect when paired with *fliE* S12R, and G129D caused a defect when paired with *fliE* Q103K (Fig. 5 and Table S3).

10.1128/mBio.02392-21.5 TABLE S3 Motility phenotypes of the different combinations of FliE and FlgB alleles as a percentage of the wild-type motility at 37°C (used in Fig. 2). Each number is an average of 8 independent assays with standard deviations given as a superscript. −, not constructed. Download Table S3, DOCX file, 0.01 MB. Copyright © 2021 Hendriksen et al. 2021 Hendriksen et al. https://creativecommons.org/licenses/by/4.0/ This content is distributed under the terms of the Creative Commons Attribution 4.0 International license.

### FliE facilitates FliE-Bla secretion into the periplasm, which is dependent on a functional fT3SS.

FliE assembly provides a structural transition that initiates proximal rod assembly on a completed core T3S apparatus (FliP_5_Q_4_R_1_) within the MS-ring (FliF). FliE also completes the T3S structure in that an *fliE* null strain exhibits an 8-fold reduction in secretion of Hook (FlgE) protein (15) and a substantial reduction in secretion of the hook-capping protein (FlgD) (20). Since FliE was required for secretion of the early class of flagellar secretion substrates, we wondered if FliE affected its own secretion. We presumed that FliE would be similar to FlgD and FlgE and be secreted as an early secretion substrate. However, the first FliE subunit assembled into the basal structure would have to be secreted by a flagellar T3S apparatus that lacks FliE (a bit of a causality dilemma). An *fliE-bla* fusion exhibited ampicillin resistance (Ap^r^) levels of 6.25 μg/ml compared to <1 μg/ml for the parent strain that lacks Bla, indicating it is secreted at significant levels into the periplasm (Table 3). Expression of a functional *fliE*^+^ gene, which fully complements a chromosomal *fliE* null mutant, from the arabinose-inducible P*_araBAD_* promoter showed a 4-fold increase in FliE-Bla secretion, increasing Ap-MIC levels 25 μg/ml. Secretion of FliE-Bla was dependent on a functional flagellar T3S apparatus: deletion of FliP and FliF reduced Ap^r^ levels to the background (MIC of <1 μg/ml). We also tested the effect of SecG on FliE-Bla secretion. Removal of SecG did not affect FliE-Bla secretion in our assay. Since removal of SecG did not affect FliE-Bla secretion, we conclude that SecG is not required for secretion at the inner membrane of either FliF or T3S apparatus proteins FliPQR and FlhAB.

**TABLE 3:** Effect of mutant type 2 (*secG*) and mutant flagellar type 3 secretion systems on FlhE-Bla and FliE-Bla secretion

Strain	Genotype	Ampicillin resistance MIC (μg/ml)						
		Trial 1	Trial 2	Trial 3	Trial 4	Trial 5	Trial 6	Most frequent
LT2	Wild type	<1	<1	<1	<1	<1	<1	<1
TH26634	*fliE-bla* Δ*araBAD*	6.25	6.25	6.25	6.25	6.25	6.25	6.25
TH26796	Para-*fliE*^+^ *fliE-bla*	25	12.5	25	25	25	25	25
TH26797	Para-*liE*^+^ *fliE-bla* Δ*secG*	25	25	25	25	25	25	25
TH26798	Para-*liE*^+^ *fliE-bla* Δ*fliP ΔfliF*	<1	<1	<1	<1	<1	<1	<1
TH26799	Para-*liE*^+^ *fliE-bla* Δ*secG ΔfliP ΔfliF*	<1	<1	<1	<1	<1	<1	<1

## DISCUSSION

Our suppression analysis generated compensatory single-amino-acid mutations alleviating motility defects caused by mutations in *fliE* in novel (*flgC*, *fliF*, *fliR*, and *flk*) and one previously described (*flgB*) site. With suppressor alleles found in (i) a component of the core secretion apparatus gene, *fliR*, (ii) the gene encoding the inner membrane ring into which the secretion apparatus assembles, *fliF*, and (iii) in two early rod substrate genes, *flgB* and *flgC*, our results support the hypothesis that FliE interacts with the MS ring, the secretion apparatus, and the FlgB and FlgC components of the proximal rod of the flagellar T3S system. This is suspected to be facilitated by protein-protein interaction involving the N and C terminus of FliE with the central portion of the protein acting as a spacer region. A portion of the C-terminal region of FliE, codons M84 to V99, is evidenced here to interact with the region of FlgB between codons G119 and G129.

The isolation of deletions of the *flk* locus that suppress the FliE Q103K substitution was unexpected. Flk is a 333-residue protein, and the sequence of the last 18 hydrophobic amino acids serves to anchor it into the cytoplasmic membrane, which is essential for its function (28). Flk has been shown to prevent the flagellar secretion specificity switch from early to late secretion prior to HBB completion (29–31). We presume that the FliE Q103K substitution is defective in secretion and removal of Flk suppresses the FliE Q103K secretion defect.

The *fliE* suppressor mutations that have been found support the hypothesis that FliE acts as a link between the rod components and the MS ring and secretion apparatus, which is now shown in the structure of the flagellar basal body (18). The data also suggest that the C terminus of FliE interacts with the C terminus of FlgB. The lack of single-amino-acid substitutions found in the middle section of the protein supports that only the N-terminal and C-terminal domains of the protein are necessary for function or secretion.

The MIC of a strain expressing an FliE-Bla fusion increased 4-fold when a wild-type *fliE* gene was expressed from the chromosomal *araBAD* promoter (Table 3). Thus, FliE facilitates its own secretion, even though the initial FliE molecules are secreted through an fT3SS lacking FliE. The *fliE* gene is the only structural component of the HBB that is transcribed in a single-gene operon. We speculate that the *fliE* gene requires an increased level of expression, either increased transcription or translation, relative to other HBB structural genes. Alternatively, mRNA signals in the 5′ or 3′ untranslated regions of the *fliE* mRNA transcript may localize *fliE* mRNA to the cytoplasmic base of the fT3SS to enhance FliE secretion.

**FIG 6:**
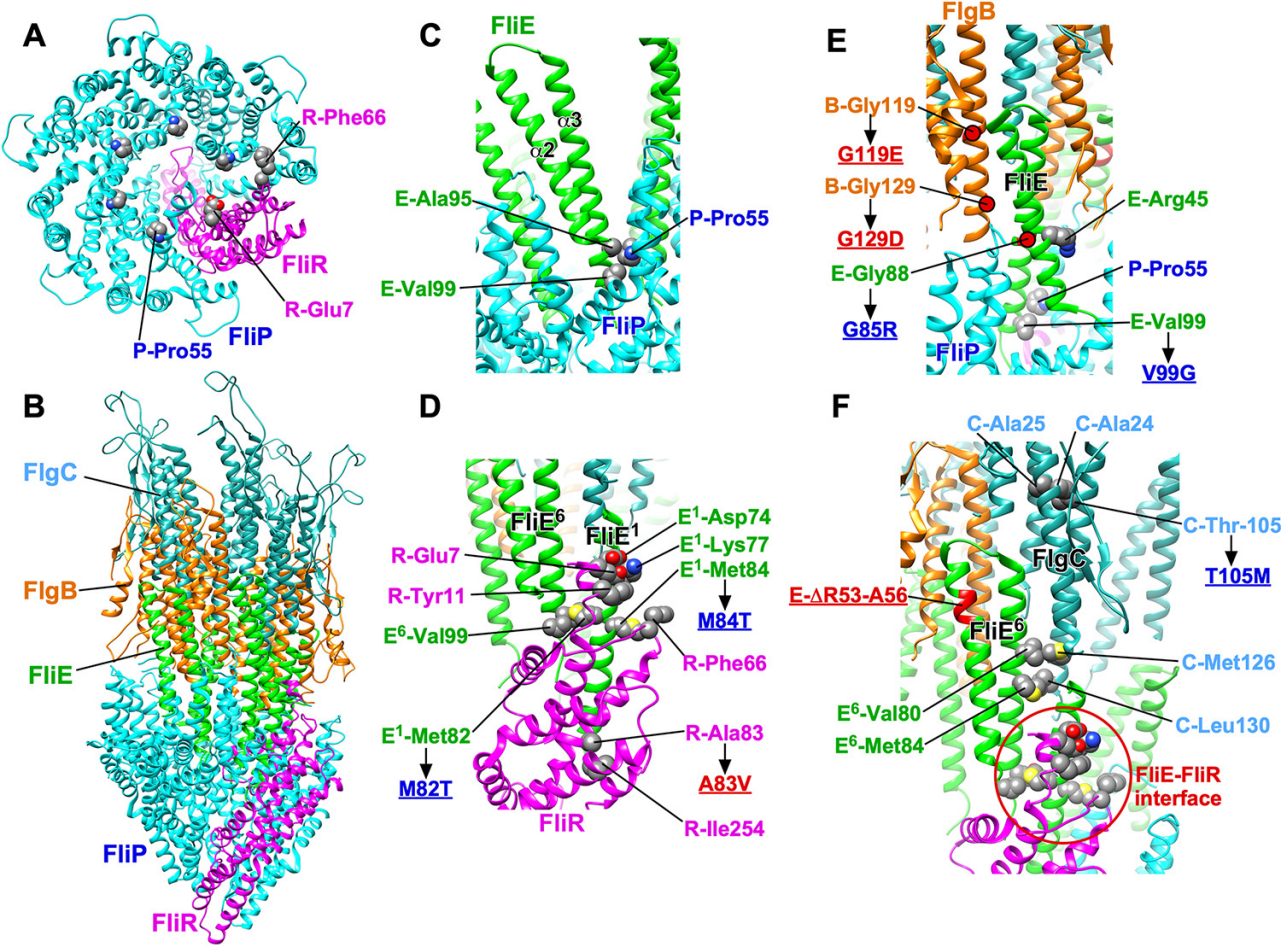
Model of intergenic suppressor alleles in *flgB*, *flgC*, *flgF*, and *fliR* with their corresponding *fliE* suppressed alleles. (A) CryoEM structure of purified FliP_5_FliQ_4_FliR_1_ complex (PDB entry 6F2D). The exit gate of the protein export channel is closed in the purified FliP_5_FliQ_4_FliR_1_ complex. FliP and FliR are shown. Pro-55 of FliP, Glu-7 of FliR, and Phe-66 of FliR, which are involved in the interaction with domain D0 of FliE, are free in the closed form. (B) CryoEM structure of export gate and rod refined in focused C1 map (7BIN). (C) Interaction between FliE and FliP. Ala-95 and Val-99 of FliE hydrophobically interact with Pro-55 of FliP. (D) Interaction between FliE and FliR. Asp74 and Lys-77 of FliE interact with Glu-7 of FliR, and the side chain of Tyr-11 of FliR stabilizes these hydrophobic and electrostatic interactions. Met-82 of FliE makes a hydrophobic contact with Val-99 of its nearest FliE subunit. Met-84 of FliE interacts with Phe-66 of FliR. The G65D/M82T/M84T triple mutation seems to weaken FliE-FliE and FliE-FliR interactions. The A83V mutation in FliR overcomes this triple mutation. (E) Interaction between FliE and FlgB. Domain D0 of FliE interacts with domain D0 of FlgB, consisting of the N-terminal and C-terminal α-helices. The FliE(G85R) mutation induces helical rearrangements of domain D0 through the interaction of this mutated residue with Arg-45 of FliE, thereby affecting the hydrophobic interaction between Val-99 of FliE and Pro-55 of FliP. The G119E and G129D mutations in FlgB restores not only the FliE(G85R) defect but also the FliE(V99G) defect. (F) Interaction between FliE and FlgC. Domain D0 of FliE also makes hydrophobic contacts with domain D0 of FlgC consisting of the N-terminal and C-terminal α-helices. Deletion of residues 53 to 56 of FliE induces helical rearrangements of domain D0 of FliE, thereby affecting not only FliE-FlgC interactions but also FliE-FliR interaction. The T105M mutation in FlgC suppresses this in-frame deletion.

The structure of the FliE within the intact flagellar basal body has recently been solved (Fig. 1) (18, 19). This allows us to assess how the *fliE* mutants and the extragenic suppressors described in this study might affect the function of FliE as an adaptor between the core fT3SS, the FliF MS-ring, and the proximal rod component of the basal body. FliE consists of three α-helices, α1, α2, and α3 (Fig. 6) (18, 19). The α1 helix binds to the inner wall of the MS-ring. Since the α2 and α3 helices form a coiled coil (domain D0) in a way similar to other rod proteins, intermolecular hydrophobic interactions of domain D0 of FliE with FliP, FliR, FlgB, and FlgC promote FliE assembly into a tubular structure with a helical symmetry. The FliE(V10G), FliE(S12R), and FliE(M19L) substitutions in helix α1 seem to affect the FliE-FliF interaction, thereby reducing normal FliE function in the fT3SS (Table 1 and Fig. 3). Most of *fliE* mutations are identified in domain D0 of FliE (Table 1 and Fig. 3), so they are likely to affect the intermolecular interactions of FliE with FliP, FliR, FlgB, or FlgC.

FliP, FliQ, and FliR form the core protein export channel of the fT3SS (Fig. 6A) (13). Interaction of FliE with the core induces a conformational change in the core to significantly enhance substrate secretion. The exit gate of purified FliP_5_FliQ_4_FliR_1_ is in a closed conformation (13). FliP, FliQ, and FliR assemble into a tubular structure with a helical symmetry similar to that of the rod, so FliE directly assembles at the distal end of the FliP_5_FliQ_4_FliR_1_ complex (Fig. 6B) (13). Domain D0 formed by α2 and α3 helices of FliE binds to FliP and FliR and induces helical rearrangements of the protein export channel, resulting in an open conformation of the exit gate (18, 19). Ala-95 and Val-99 of FliE make a hydrophobic contact with Pro-55 of FliP (Fig. 6C). The FliE(V99G) mutation inhibits the secretion of FlgD into the periplasm (20), suggesting that FliE with the V99G mutation does not open the exit gate of the protein export channel efficiently. Therefore, we propose that these hydrophobic interactions of FliE with FliP are critical not only for the assembly of FliE into the most proximal part of the rod but also for efficient opening of the exit gate of the protein export channel.

FliE also interacts with FliR in the basal body (Fig. 6D). Lys-77 and Asp74 of FliE interact with Glu-7 of FliR, and a hydrophobic contact of the side chain of Tyr-11 of FliR with that of Glu-7 of FliR could stabilize FliE-FliR interactions. Met-84 of FliE is also directly involved in the interaction with Phe-66 of FliR. Met-82 of FliE interacts with Glu-98 and Val-99 of its neighboring FliE subunit. The FliE(G65D/M82T/M84T) triple mutation inhibits flagellar assembly, but neither G65D, M82T, nor M84T affect flagellum-driven motility. We presume that the triple mutation weakens not only FliE-FliE intermolecular interaction but also FliE-FliR interactions. The FliR(A83V) suppressor mutation restored motility of the triple mutant to a significant degree. The A83V mutation is quite far from the FliE-FliR interface. Val-83 is likely to make a hydrophobic contact with Ile-254 of FliR, presumably causing a remodeling of hydrophobic side chain interaction networks in FliR. As a result, FliE(G65D/M82T/M84T) can bind to FliR to assemble into a helical structure at the tip of the FliP_5_FliQ_4_FliR_1_ complex. Therefore, we propose that the binding of FliE to FliR induces the remodeling of hydrophobic interaction networks in FliR, allowing FliR to change its conformation from the closed form to the open one.

The FliE(G85R) substitution significantly reduced motility at 37°C. This mutation could affect an interaction between helices α2 and α3 because of its proximity to Arg-44 in the α2 helix and induce helical rearrangements of domain D0. The FlgB(G119E) and FlgB(G129D) suppressor mutations overcome not only the FliE(V99G) defect but also the FliE(G85R) defect, suggesting that the G85R mutation affects FliE-FliP interactions in a way similar to that of FliE(V99G) (20). Because the FlgB(G119E) and FlgB(G129D) suppressor mutations are located in domain D0 of FlgB, we suggest that these two mutations affect the FliE-FlgB interface, allowing either FliE(V99G) or FliE(G85R) to stably associate with FliP. Therefore, we propose that intermolecular packing interactions between domains D0 of FliE and FlgB stabilize the FliE-FliP interactions.

In-frame deletion of residues 53 to 56 significantly reduced motility at both 30°C and 37°C. This deletion changes spacing between helices α2 and α3, affecting hydrophobic interaction networks formed by Val-80 of FliE, Met-84 of FliE, Met-126 of FlgC, and Leu-130 of FlgC. The FlgC(T105M) suppressor mutation significantly improved the motility of the *fliE*(*ΔR53-A56*) mutant. FlgC Met-105 seems to make hydrophobic contacts with both Ala-24 and Ala-25 of FlgC, thereby changing an orientation of the C-terminal α-helix containing Met-126 of FlgC and Leu-130 of FlgC. This could allow the T105M substitution in domain D0 of FlgC to interact with domain D0 of FliE so that FlgC can assemble above FliE. Because the deletion reduces the probability of flagellar assembly considerably (Table 1 and Fig. 3), we propose that intermolecular packing interactions between domains D0 of FliE and FlgC also stabilize FliE-FliR interactions.

The FliE protein has a unique role in the assembly and function of the bacterial flagellum. The isolation and characterization of mutants and extragenic suppressors described here, in combination with the resolution of its structure within the intact basal body, provides insight as to its role as an adaptor between the proximal rod at FlgB and FlgC, the MS-ring, and the core secretion pore complex (FliP_5_FliQ_4_FliR_1_). Furthermore, the final assembly of FliE into the basal structure allows for a conformational transition in the secretion core of fT3SS to optimize secretion at rates of thousands of amino acids per second for the axial components that make up the rod, the hook, and the long external filament. To our knowledge, there is no other secretion system in biology capable of such high rates of protein secretion.

## MATERIALS AND METHODS

### Bacterial strains and media.

All bacterial strains used in this study are derived from Salmonella Typhimurium strain LT2 and are listed in Table S1 in the supplemental material. Lysogeny broth (LB) contained 10 g of Bacto-tryptone (Difco), 5 g of yeast extract, 5 g of NaCl per liter. Soft agar motility plates contained 10 g of Bacto-tryptone, 5 g of NaCl, and 3 g of Bacto-agar (Difco) per liter. Tetracycline-sensitive selection medium (32) was modified to replace chlortetracycline with anhydrotetracyline (A-Tc), added after medium was autoclaved, and cooled just prior to pouring, to a final concentration of 0.5 ng/ml. l-Arabinose was added to a final concentration of 0.2%. Unless indicated otherwise, antibiotics were added as described (27).

10.1128/mBio.02392-21.3 TABLE S1 List of strains used in this study. Download Table S1, DOCX file, 0.01 MB. Copyright © 2021 Hendriksen et al. 2021 Hendriksen et al. https://creativecommons.org/licenses/by/4.0/ This content is distributed under the terms of the Creative Commons Attribution 4.0 International license.

### Strain construction.

The generalized transducing phage P22 *HT105/1 int-201* was used for all transductional crosses (27). Targeted chromosomal mutagenesis was performed via the *tetRA* cassette insertion-replacement method using the λ-Red recombination system as described previously (23). For *fliE*, coding sequences for selected 17-amino-acid long sections were targeted by *tetRA* insertion-deletion and replacement with doped oligonucleotides (Table S1). Doped oligonucleotides were ordered to contain one base pair mutation within each targeted region (Table S2). Targeted mutagenesis of *fliE* was performed in a genetic background containing a Mu*d*-*lac* operon fusion to the *fljB* flagellin gene (*fljB*::Mu*d*J). In this background, cells are Lac^+^ if FliE is functional and Lac^−^ if FliE is defective (33). Intermediate levels of FliE function were screened for with tetrazolium-lactose indicator media (22, 28). The activity of FliE following mutagenesis was assessed using both motility and *fljB*::Mu*d*J lactose activity assays (Fig. 2). Mutants with intermediate motility and/or *fljB*::Mu*d*J expression levels were then characterized by DNA sequence analysis.

10.1128/mBio.02392-21.4 TABLE S2 List of primers used in this study. The uppercase letters in the sequences denotes the oligonucleotide-directed mutagenized region of *fliE*. Download Table S2, DOCX file, 0.01 MB. Copyright © 2021 Hendriksen et al. 2021 Hendriksen et al. https://creativecommons.org/licenses/by/4.0/ This content is distributed under the terms of the Creative Commons Attribution 4.0 International license.

The construction of C-terminal β-lactamase fusions to FliE (FliE-Bla) was done by insertion of a *tetRA* element before the *fliE* stop codon. The *tetRA* element was then replaced via λ-Red recombination with a DNA sequence that included the *bla* coding sequence lacking its first 23 amino acid codons, which remove the Sec secretion signal for Bla.

### Random mutagenesis of the *fliE* gene.

A *tetRA* cassette was inserted, by λ-Red recombination, 2 bases upstream of the *fliE* ATG start codon, resulting in a *tetA-fliE* operon fusion with both *tetA* and *fliE* expressed from the *tetA* promoter (P*_tetA_*) (strain TH4756). This strain is motile only under P*_tetA_* inducing conditions, which includes the addition of Tc, chlortetracycline, or A-Tc to the growth medium. Note that A-Tc serves as an inducer of P*_tetA_* transcription but, unlike Tc, is not an antibiotic. The *tetA* gene in TH4756 was then deleted again by λ-Red recombination to generate a P*_tetA_*-fliE^+^-containing strain (TH11299), which was Tc^s^ and motile in the presence of A-Tc.

The *fliE* coding region of TH4756 was mutagenized by error-prone PCR as previously described (34) using fliE60B and tetAfliE primers. PCR was performed for 30 cycles using HOT start *Taq* polymerase (Qiagen). A second PCR using proofreading *Taq* polymerase (Ecuzyme) with primers fliE60B and tetAfliElinker was performed on the mutagenized *fliE* DNA to provide flanking DNA for λ-Red recombination. The PCR product was purified by agarose gel electrophoresis and the correct size DNA fragment eluted for electroporation into TH11299 expressing λ-Red recombinase from plasmid pKD46 (35), selecting for Tc^r^ and screening for a nonmotile phenotype on motility plates with added Tc.

### Structure modeling.

To understand how extragenic suppressors in FlgC, FlgB, and FliR restore the assembly of FliE at the distal end of the export gate complex, the molecular modeling system UCSF Chimera (https://www.cgl.ucsf.edu/chimera/) was used to display cryoEM structures of the export gate complex with (PDB entry 7BIN) or without (PDB entry 6F2D) the proximal rod.
