# Supplementary material for: Genetic Analysis of the Salmonella FliE Protein That Forms the Base of the Flagellar Axial Structure
Source: mBio. 2021 Sep 28;12(5):e02392-21. doi: 10.1128/mBio.02392-21 (PMC8546590; doi:10.1128/mBio.02392-21)
Supplement: TABLE S3 [file mbio.02392-21-st003.docx]

**Supplementary Table 3:** Motility phenotypes of the different combinations of FliE and FlgB alleles as a percentage of the wildtype motility at 37^o^C (used in Figure 2). Each number is an average of 8 independent assays with standard deviation given as superscript. (“-“ = not constructed).

|  | *flgB* Allele | | |
| --- | --- | --- | --- |
| *fliE* Allele | *flgB*^+^ | G119E | G129D |
| V10G | 52^±7^ | 54^±6^ | 47^±8^ |
| S12R | 66^±9^ | 53^±9^ | 68^±6^ |
| ∆Q37 | 30^±3^ | 35^±4^ | - |
| G62V | 75^±9^ | 71^±4^ | 85^±8^ |
| M84K | 75^±8^ | 75^±9^ | 86^±9^ |
| G85R | 33^±14^ | 71^±16^ | 85^±4^ |
| V88G | 50^±19^ | 81^±5^ | 77^±11^ |
| V88E | 19^±7^ | 43^±18^ | 62^±16^ |
| K91N | 81^±6^ | 79^±8^ | 80^±7^ |
| V93G | 59^±11^ | 70^±9^ | 67^±6^ |
| A95G | 89^±11^ | - | 80^±8^ |
| Y96C | 45^±14^ | 46^±6^ | 70^±13^ |
| M102R | 5^±1^ | 5^±2^ | 6^±3^ |
| Q103K | 27^±2^ | 28^±3^ | 24^±2^ |
| *fliE*^+^ | 100 | 89^±6^ | 65^±12^ |
