# Supplementary material for: Genetic Analysis of the Salmonella FliE Protein That Forms the Base of the Flagellar Axial Structure
Source: mBio. 2021 Sep 28;12(5):e02392-21. doi: 10.1128/mBio.02392-21 (PMC8546590; doi:10.1128/mBio.02392-21)
Supplement: TABLE S1 [file mbio.02392-21-st001.docx]

__________________________________________________________________________

Strain Genotype Source*

TH437 wild type LT2 John Roth

TH4756 *fliE5535*::*tetRA*

TH7365 *fljB5001*::Mu*d*J Δ*hin-5718*::FRT

TH11299 Δ*fliE7050*::*tetRA* Δ*tetA3501*

TH11303 *fliE5535*::*tetRA* *fliE7053*(D74G S79P)

TH11305 *fliE5535*::*tetRA fliE7055*(G65D M82T M84T)

TH11373 *fliE5535*::*tetRA* *fliE7053* *zea-3532*::Tn*10d*Cm *fliF7059*(N209D)

TH11374 *fliE5535*::*tetRA* *fliE7055* *zea-3532*::Tn*10d*Cm *fliR7060*(A83V)

TH23090 Δ*fliE8480*::*tetRA*(ΔAA2-19) *fljB5001*::Mu*d*J Δ*hin-5718*::FRT

TH23091 Δ*fliE8481*::*tetRA*(ΔAA87-104) *fljB5001*::Mu*d*J Δ*hin-5718*::FRT

TH23581 Δ*fliE8509*::*tetRA*(ΔAA20-37) *fljB5001*::Mu*d*J Δ*hin-5718*::FRT

TH23583 Δ*fliE8510*::*tetRA*(ΔAA38-55) *fljB5001*::Mu*d*J Δ*hin-5718*::FRT

TH23585 Δ*fliE8511*::*tetRA*(ΔAA56-73) *fljB5001*::Mu*d*J Δ*hin-5718*::FRT

TH23587 Δ*fliE8512*::*tetRA*(ΔAA74-86) *fljB5001*::Mu*d*J Δ*hin-5718*::FRT

TH24121 *flgC8647*(T105M) *fliE8646*(Δ(AA53-56)) *fljB5001*::Mu*d*J Δ*hin-5718*::FRT

TH24275 Δ*fliE8512*::*tetRA* DUP*4126*[*fliF*(AA126)*Km^R^**amyA*(AA166)]

TH25966 *fliE8513*(V10G) *fljB5001*::Mu*d*J Δ*hin-5718*::FRT

TH25967 *fliE8881*(S12R) *fljB5001*::Mu*d*J Δ*hin-5718*::FRT

TH25968 *fliE8882*(M19L) *fljB5001*::Mu*d*J Δ*hin-5718*::FRT

TH25969 *fliE8883*(ΔQ37) *fljB5001*::Mu*d*J Δ*hin-5718*::FRT

TH25970 *fliE8646*(ΔR53-A56) *fljB5001*::Mu*d*J Δ*hin-5718*::FRT

TH25971 *fliE8884*(G62V) *fljB5001*::Mu*d*J Δ*hin-5718*::FRT

TH25972 *fliE8885*(I66T) *fljB5001*::Mu*d*J Δ*hin-5718*::FRT

TH25973 *fliE8886*(A56A A67P) *fljB5001*::Mu*d*J Δ*hin-5718*::FRT

TH25974 *fliE8887*(L68F) *fljB5001*::Mu*d*J Δ*hin-5718*::FRT

TH25975 *fliE8888*(S79P) *fljB5001*::Mu*d*J Δ*hin-5718*::FRT

TH25976 *fliE8889*(V80A) *fljB5001*::Mu*d*J Δ*hin-5718*::FRT

TH25977 *fliE8890*(M84K) *fljB5001*::Mu*d*J Δ*hin-5718*::FRT

TH25978 *fliE7066*(M84T) *fljB5001*::Mu*d*J Δ*hin-5718*::FRT

TH25979 *fliE8891*(G85R) *fljB5001*::Mu*d*J Δ*hin-5718*::FRT

TH25980 *fliE8892*(V88E) *fljB5001*::Mu*d*J Δ*hin-5718*::FRT

TH25981 *fliE8893*(V88G) *fljB5001*::Mu*d*J Δ*hin-5718*::FRT

TH25982 *fliE8894*(N90H) *fljB5001*::Mu*d*J Δ*hin-5718*::FRT

TH25983 *fliE8895*(K91N) *fljB5001*::Mu*d*J Δ*hin-5718*::FRT

TH25984 *fliE8896*(V93I) *fljB5001*::Mu*d*J Δ*hin-5718*::FRT

TH25985 *fliE8897*(V93G) *fljB5001*::Mu*d*J Δ*hin-5718*::FRT

TH25986 *fliE8898*(A95G) *fljB5001*::Mu*d*J Δ*hin-5718*::FRT

TH25987 *fliE8899*(A95S) *fljB5001*::Mu*d*J Δ*hin-5718*::FRT

TH25988 *fliE8900*(Y96C) *fljB5001*::Mu*d*J Δ*hin-5718*::FRT

TH25989 *fliE8901*(S101F) *fljB5001*::Mu*d*J Δ*hin-5718*::FRT

TH25990 *fliE8591*(M102R) *fljB5001*::Mu*d*J Δ*hin-5718*::FRT

TH25991 *fliE8902*(V104A) *fljB5001*::Mu*d*J Δ*hin-5718*::FRT

TH25992 *flgB8903*(G119E) *fliE8891*(G85R) *fljB5001*::Mu*d*J Δ*hin-5718*::FRT

TH25993 *flgB8904*(G129D) *fliE8891*(G85R) *fljB5001*::Mu*d*J Δ*hin-5718*::FRT

TH25994 *flk-8905*(W54stopUAG) *fliE8489*(Q103K) fljB *fljB5001*::Mu*d*J Δ*hin-5718*::FRT

TH25995 *flk-8906*(Q208stopUAG) *fliE8489*(Q103K) *fljB5001*::Mu*d*J Δ*hin-5718*::FRT

TH25996 Δ*pdxB*-*flk-8907* *fliE8489*(Q103K) *fljB5001*::Mu*d*J Δ*hin-5718*::FRT

**Supplementary Table 1.** List of strains used in this study.

*Unless indicated otherwise, these strains were constructed during the course of this work.
