# Supplementary material for: Genetic Analysis of the Salmonella FliE Protein That Forms the Base of the Flagellar Axial Structure
Source: mBio. 2021 Sep 28;12(5):e02392-21. doi: 10.1128/mBio.02392-21 (PMC8546590; doi:10.1128/mBio.02392-21)
Supplement: TABLE S2 [file mbio.02392-21-st002.docx]

| Primer Name | Sequence |
| --- | --- |
| 1676-fliE3amp | cgcccgggagttcgctgcggatgtgttgtcagccctggcttaccaatgcttaatcagtga |
| 2342-bla-fliEwt | gatcttcagcatcttttactttcaccagcgtttctgggtgaacctgcatggacatgactt |
| 7292-fliE21-tetR | aggggttattagccagttacaggcgacggcgatggccgcgttaagacccactttcacatt |
| 7293-fliE28tetA | cagcgcagcatgtagctggccggcaaaactcaccgtcgactgctaagcacttgtctcctg |
| 7294-delfliE-aa18-31fd | gcagcaatacaggggattgaaggggttattagccagttacaggcgacggtgagttttgcc |
| 7295-delfliEaa15-28 | tggcagcaatacaggggattgaaggggttattagccagttacagtcgacggtgagttttg |
| 7296-delfliEaa21-34 | attgaaggggttattagccagttacaggcgacggcgatggccgccggccagctacatgct |
| 7323-fliE34 fillin | tgtcgctaatacgatcca |
| 7352-fliEAA2tetR | aatctctgctgcgtaaaaaatcatccaggggaatagcatgttaagacccactttcacatt |
| 7353-fliEAA19tetA | actcaccgtcgactgcgagtgcgtgtcttgcccgcgcgcggcctaagcacttgtctcctg |
| 7354-fliE87tetR | cgatatgcaaaaagcgtccgtctctatgcaaatggggattttaagacccactttcacatt |
| 7355-fliE104tetA | cgcccgggagttcgctgcggatgtgttgtcagccctggcttactaagcacttgtctcctg |
| 7362-fliE2-19-2%dope | aatctctgctgcgtaaaaaatcatccaggggaatagcatgGCAGCAATACAGGGGATTGAAGGGGTTATTAGCCAGTTACAGGCGACGGCGATGgccgcgcgcgggcaagacacgcactcgcagtcgacggtga |
| 7363-fliE2-19fill | tcaccgtcgactgcgagt |
| 7364-fliE87-104-2%dope | cgatatgcaaaaagcgtccgtctctatgcaaatggggattCAGGTGCGCAACAAGCTGGTCGCCGCGTATCAGGAAGTCATGTCCATGCAGGTTtaagccagggctgacaacacatccgcagcgaactcccggg |
| 7365-fliE87-104fill | cccgggagttcgctgcgg |
| 7366-fliE2-19PCRcheck | ttatttaacgcctttatctt |
| 7437-fliEAA20-37tetA | ccgcttgtctgtcgctaatacgatccagcgcagcatgtagctaagcacttgtctcctg |
| 7438-fliEAA20-37tetR | ggattgaaggggttattagccagttacaggcgacggcgatttaagacccactttcacatt |
| 7439-fliEAA20-37-fillin | ccgcttgtctgtcgctaa |
| 7440-fliEAA20-37doped | ggattgaaggggttattagccagttacaggcgacggcgatGGCCGCGCGCGGGCAAGACACGCACTCGCAGTCGACGGTGAGTTTTGCCGGCCAGctacatgctgcgctggatcgtattagcgacagacaagcgg |
| 7441-fliEAA38-55tetR | agacacgcactcgcagtcgacggtgagttttgccggccagttaagacccactttcacatt |
| 7442-fliEAA38-55tetA | taagcgcaatgcccggctcacccagagtgaatttttccgcctaagcacttgtctcctg |
| 7443-fliEAA38-55doped | agacacgcactcgcagtcgacggtgagttttgccggccagCTACATGCTGCGCTGGATCGTATTAGCGACAGACAAGCGGCGGCGCGCGTTCAGgcggaaaaattcactctgggtgagccgggcattgcgctta |
| 7444-fliEAA38-55-fillin | taagcgcaatgcccggct |
| 7445-fliEAA56-73tetR | ggatcgtattagcgacagacaagcggcggcgcgcgttcagttaagacccactttcacatt |
| 7446-fliEAA56-73tetA | gaatccccatttgcatagagacggacgctttttgcatatcctaagcacttgtctcctg |
| 7449-fliEAA74-86tetR | tctgggtgagccgggcattgcgcttaatgacgtgatggccttaagacccactttcacatt |
| 7450-fliEAA74-86tetA | tgacttcctgatacgcggcgaccagcttgttgcgcacctgctaagcacttgtctcctg |
| 7509-fliEAA20-37-fillin-rev | ggattgaaggggttatta |
| 7510-fliEAA38-55-fillin-rev | agacacgcactcgcagtc |
| 7511-fliEAA74-86doped | gcttaatgacgtgatggccGATATGCAAAAAGCGTCCGTCTCTATGCAAATGGGGATTcaggtgcgcaacaagctgg |
| 7512-fliEAA74-86-revhomology | ttcactctgggtgagccgggcattgcgcttaatgacgtgatggc |
| 7513-fliEAA74-86-fwdhomology | ggacatgacttcctgatacgcggcgaccagcttgttgcgcacct |
| 7655-DfliE37-41-clean | gaacgcgcgccgccgcttgtctgtcgctaatacgatccaggccggcaaaactcaccgt |
| 7656-DfliE37-41-fill | cgcgggcaagacacgcactcgcagtcgacggtgagttttgccggc |
| 7657-fliEAA37-41-change | cgcgcgccgccgcttgtctgtcgctaatacgatccagNNNNNNNNNNNNNNNgccggcaaaactcaccgtcgactgcgagtgcgtgtct |
| 7973-fliEAA85-89-change | ttgcgcttaatgacgtgatggccgatatgcaaaaagcgtccgtctctatgcaaatgNNNNNNNNNNNNNNNaacaagctggtcgccgcg |
| 7974-fliEAA85-89-fillin | tcagccctggcttaaacctgcatggacatgacttcctgatacgcggcgaccagcttgtt |
| 8260-fliE-clean | gtaaaaaatcatccaggggaatagcatggcagcaatacagtccatgcaggttTAAgcc |
| 8326-fliE55-73-fwd-fill | ggatcgtattagcgacagacaagcggcggcgcgcgttcag |
| 8327-fliE55-73-rev-fill | gaatccccatttgcatagagacggacgctttttgcatatc |
| 8328-fliEAA55-73Doped | gcggcggcgcgcgttcagGCGGAAAAATTCACTCTGGGTGAGCCGGGCATTGCGCTTAATGACGTGATGGCCgatatgcaaaaagcgtcc |

**Supplementary Table 2.** List of primers used in this study. The uppercase letters in the sequences denotes the oligonucleotide-directed mutagenized region of *fliE*.
